# The study on the association between Beijing genotype family and drug susceptibility phenotypes of *Mycobacterium tuberculosis* in Beijing

**DOI:** 10.1038/s41598-017-14119-z

**Published:** 2017-11-08

**Authors:** Yi Liu, Xiaoying Jiang, Wensheng Li, Xuxia Zhang, Wei Wang, Chuanyou Li

**Affiliations:** Department of Bacteriology and Immunology, Beijing Key Laboratory on Drug-Resistant Tuberculosis Research, Beijing Tuberculosis and Thoracic Tumor Research Institute/Beijing Chest Hospital, Capital Medical University, Tongzhou District, Beijing, 101149 PR China

## Abstract

The predominant prevalent *Mycobacterium tuberculosis* (*M*. *tb*) lineage was the Beijing genotype family in Beijing. There has been no systematic study on the association between drug resistance and Beijing genotype. Here we collected 268 *M*. *tb* strains, analyzed the background information and the bacteriological characteristics. The mean age of the cases was 40.12 years; male patients were almost three times than female patients. After genotyping analyzation, 81.7% (219/268) strains were categorized as Beijing genotype; no significant difference was observed between Beijing and non-Beijing genotype in gender, age and treatment history. Drug susceptibility testing (DST) analyzation demonstrated that 172 (64.2%) strains were fully sensitive to all drugs (Isoniazid, Rifampin, Streptomycin, and Ethambutol), while 96 (35.8%) strains were resistant to at least one of the drugs. Beijing genotype strains exhibited a significantly higher clustering rate. However, no significant association relationship was observed between drug resistance and Beijing genotype family. The study provided insights into the genotype diversity and revealed that the frequencies of drug-resistance of Beijing genotype strains.It would be helpful for the establishment of the efficient tuberculosis (TB) prevention and control strategy in Beijing.

## Introduction

Tuberculosis (TB), caused by *Mycobacterium tuberculosis* (*M*. *tb*) complex, is one of the major threats to the public health around the world and China^1^. Prevention and control of TB are also confronting with more pressure in Beijing due to the rapid urbanization and economic transition^2,3^.

The molecular epidemiology methods has been proven playing an important role in TB control and study^4,5^. Among these, spoligotyping has been considered as a powerful tool to differentiate *M*. *tb* complex into various genotypes, especially for identifying Beijing genotype^6^. Similarly, variable number of tandem repeat (VNTR) genotyping has also been considered as a much better method, as it faster and easier to perform, as well as possesses higher discriminatory power. Both of them can be used friendly and easily compared through generating numerical data. Moreover, the combined application of spoligotyping and VNTR is increasingly common in *M*. *tb* molecular epidemiology research^7,8^.

The molecular typing of *M*. *tb* has greatly improved knowledge of TB epidemiology and enabled molecular guided control of the disease. Based on the molecular epidemiology technologies, some studies shown that drug resistance of *M*. *tb* was associated with genotype diversity^6,9–11^. However, other studies have not indicated the association relationship between them^12,13^. It is possible that the results were changed according to the project setting or drug types^14^. Previous reports^15–17^ and our study^18^ have demonstrated that Beijing genotype strains were highly prevalent in Beijing, occupied around 82%. However, there has been no systematic study on the association between drug resistance and Beijing genotype. It is quite necessary to clarify the situation in Beijing considering the high tuberculosis prevalence and non-consistent results acquired from different regions and researchers.

In order to clarify the association relationship, a retrospective study was carried out on the genotype diversity and the frequencies of drug-resistance of 268 *M*. *tb* strains. We genotyped strains collected from Beijing by spoligotyping and VNTR technology. Additionally, the background information and DST profiles of these cases were also analyzed. The study was conducted to gain insights into the genotype characteristics of *M*. *tb* strains and the association with drug resistance. It would be helpful for developing better TB prevention and control strategies in Beijing.

## Results

### Study population

**Figure 1:**
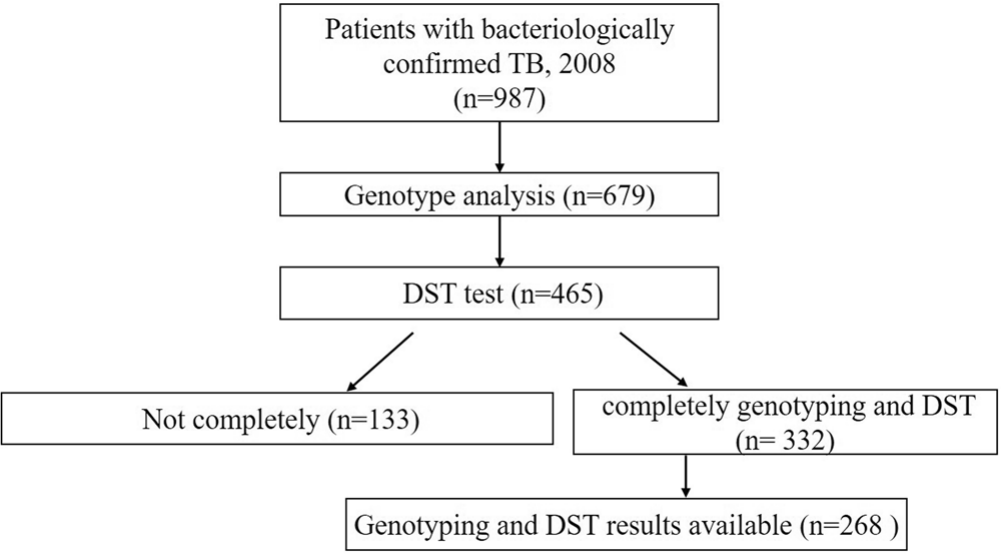
Study population of cases of tuberculosis and study flowchart in this study.

To investigate the characteristics of the study population, we analyzed the basic information of 268 individual strains collected from Beijing in 2008(Fig. 1). Classification of all isolates investigated in this study according to patients’ age and gender were described in Table 1.The enrolled patients included 206 males (76.9%) and 62 females (23.1%), male to female ratio was 3.32; the mean age was 40.12 years old (range from 11 to 89, med 38). Of them, 147 patients were Beijing residents and 121 were migrant population (non-residents) (Table 1). It indicated that the resident population and non-resident population contributed the same to TB prevalence. Additional, we did not find a significant difference between Beijing and non-Beijing genotype strains when considering risk factors such as gender, age, registered residence and treatment history.

**Table 1:** The background information of 268 patients.

Characteristic	Variable	Number of reported cases (%)	Beijing family (%)	Non-Beijing Family (%)	OR (95% CI)	*p* Value
All		268	219	49		
Sex	Male	206 (76.9)	169 (77.2)	37 (75.5)	1.022 (0.858–1.217)	0.402
	Female	62 (23.1)	50 (22.8)	12 (2.5)		
Age groups, years	<44	166 (61.9)	137 (62.6)	29 (159.2)	1.031 (0.802–1.325)	0.405
	≥44	102 (38.1)	83 (37.9)	19 (38.8)		
Household	City	147 (54.9)	121 (55.3)	26 (53.1)	0.970 (0.825–1.141)	0.362
	migrant population	121 (45.1)	98 (44.7)	23 (47)		
History of TB	No Treatment	207 (77.2)	169 (77.2)	38 (77.6)	1.041 (0.780–1.390)	0.39
	Treatment	61 (22.8)	51 (23.2)	10 (20.4)		

*P* value indicates whether there is a significant difference between Beijing family and non-Beijing family in sex, age, household, and history of TB. (*P* < 0.05 represents a statistically significant difference).

### The spoligotyping and VNTR genotyping structure of *M*. *tb* strains

To clarify the genotype diversity of prevalent strains in Beijing, we analyzed 268 *M*. *tb* strains using spoligotyping and VNTR method. Among these strains, 219 (81.7%) were belonged to Beijing genotype families and 49 (18.3%) were belonged to non-Beijing genotype families (see Supplementary Table S1). These results indicated that Beijing genotype strains were the most prevalent strains in Beijing. Of all Beijing-family strains, typical Beijing families (SIT1) accounted for 198 (74.2%), while atypical Beijing families accounted for 21 (7.9%). Among non-Beijing family strains, mainly included T1 (SIT53) family, T2 (SIT52) family, MANU2 (SIT54) family, and H3 (SIT50) family *et al*. furthermore 16 (5.29%) strains constituted a newly found genotype cluster.

To analyze the above results from clustering angle and demonstrate the genetic linkage map, we made a minimum spanning tree (MST) based on spoligotyping data by the BioNumerics software. MST demonstrated that these strains clustered by a graph. Meanwhile, we calculated the genotypes of the strains using locus variant counts and distances. As shown in see Supplementary Fig. S1, the largest cluster containing 198 strains (SIT1) belonged to the typical Beijing family and was surrounded by small clusters of the atypical Beijing family. Beijing family and non-Beijing family were divided into two main groups. The three larger clusters on the left were the T1, MANU2 family and 16 newfound genotypes surrounded by other genotypes.

The allelic diversity for the 268 strains of each VNTR locus was estimated using the Hunter-Gaston discriminatory index (HGDI) (Table 2). The VNTR 12-locus (BJ) method differentiated the 268 strains into 196 genotypes. A total of 156 strains had unique patterns and the remaining 112 formed 8 clusters (2 to 20 strains per cluster). The 12 VNTR loci exhibited different HGDI scores, ranging from 0.1423 to 0.7255. The cumulative discriminatory power (HGDI) of the total loci set reached 0.998 for Beijing family, HGDI reached 0.999 when applied to all strains. The spoligotyping method differentiated the same strains into 33 genotypes. A total of 28 strains had unique patterns and the remaining 240 formed 40 clusters (2 to 188 strains per cluster).VNTR can separate the Beijing family into different small branches and more less clusters, indicating that it possessed a higher discriminatory power than spoligotyping. The Beijing genotype family strains exhibited a significantly higher clustering rate than the non-Beijing genotype family strains by both spoligotyping and VNTR typing analyzation (Table 3), suggesting transmission of Beijing genotype should be higher than the non-Beijing genotype.

**Table 2:** The Hunter-Gaston discriminatory index of the 12 VNTR loci in M. tuberculosis strains from Beijing.

Order	VNTR locus	VNTR alias	No. of alleles	Allelic diversity (h*) for					
				Beijing family(n = 219)	Cumulative	Non-Beijing family(n = 49)	Cumulative	All strains (n = 268)	Cumulative
1	0424	Mtub04	7	0.6138	0.6138	0.6438	0.6438	0.6209	0.6209
2	1955	Mtub21	5	0.5962	0.7482	0.6765	0.7871	0.5639	0.7586
3	2074	Mtub24	5	0.4929	0.8197	0.4609	0.8375	0.4985	0.8282
4	2163	QUB-11b	7	0.6255	0.9167	0.7865	0.9281	0.7255	0.9216
5	4156	QUB-4156c	7	0.2138	0.9319	0.2970	0.9372	0.2390	0.9327
6	0960	MIRU10	6	0.5576	0.9885	0.6723	0.9896	0.6358	0.9890
7	1644	MIRU16	7	0.5782	0.9912	0.6036	0.9964	0.5523	0.9942
8	3007	MIRU20	3	0.2534	0.9933	0.2886	0.9967	0.2605	0.9945
9	2531	MIRU23	3	0.1465	0.9939	0.1490	0.9971	0.1423	0.9954
10	3192	MIRU31	8	0.3931	0.9968	0.6131	0.9990	0.5046	0.9988
11	4348	MIRU39	5	0.3082	0.9981	0.5729	0.9993	0.3961	0.9990
12	0802	MIRU40	7	0.2735	0.9983	0.6607	0.9995	0.4871	0.9992

*h, Hunter-Gaston discriminatory index.

**Table 3:** Comparison of clustered and individual strains between the Beijing and non-Beijing family.

Genotyping	Genotype	Number of reported cases (n = 268)	Beijing family(n = 219)	Non-Beijing family(n = 49)	*p* value
Spoligotyping	Individual strains, n	28	5	23	<0.001
	Clustered strains, n	240	214	26	
	Clusters, n	8	3	5	
	Clustering rate, %	90.29	97.72	57.14	
VNTR	Individual strains, n	156	120	36	0.009
	Clustered strains, n	112	97	15	
	Clusters, n	40	34	6	
	Clustering rate, %	41.79	45.21	28.57	

(*p* < 0.05 represents a statistically significant difference).

### Drug resistance patterns difference and the association with Beijing genotype family

To investigate the profile of drug-resistance of these prevalent strains, drug susceptibility testing (DST) was carried on among the 268 isolated strains. The results showed that 172 isolates (64.2%) were sensitive to the four first-line anti-TB drugs tested and 96 isolates (35.8%) were resistant to at least one of these drugs, including the 44 (16.4%) MDR strains (Table 4).

**Table 4:** The different profiles of drug susceptibility among the different genotype family and different treated cases (n = 268).

Characteristic	Category	Number of reported cases (%)	different genotype family				new and retreated cases			
			Beijing family (%)	Non-Beijing Family (%)	OR (95% CI)	*p* Value	New cases (%)	Retreated cases (%)	OR (95% CI)	*p* Value
All		268	219	49			207	61		
DST profile	Pansusceptible	172(64.2)	138(63)	34(69.4)			154(74.4)	18(29.5)	2.52(1.69–3.75)	<0.001
	INH	66(24.6)	57(26)	9(18.4)	1.47(0.75–2.66)	0.13	30(14.5)	36(59)	0.24(0.16–0.36)	<0.001
	RIF	51(19)	42(19.2)	9(18.4)	1.04(0.54–2.00)	0.448	23(11.1)	28(45.9)	0.24(0.15–0.39)	<0.001
	SM	64(23.9)	54(24.7)	10(20.4)	1.21(0.66–2.20)	0.264	37(17.9)	27(44.3)	0.40(0.27–0.606)	<0.001
	EMB	18(6.7)	15(6.8)	3(6.1)	1.12(0.33–3.72)	0.427	4(1.9)	14(23)	0.08(0.03–0.25)	<0.001
	MDR	44(16.4)	37(16.9)	7(14.3)	1.18(0.56–2.49)	0.328	18(8.7)	26(42.6)	0.20(0.12-0.35)	<0.001

INH: Isoniazid; RIF: Rifampicin; SM: Streptomycin; EMB: Ethambutol; MDR: multi-drug resistance. *p* value indicates whether there is a significant difference between Beijing family and non-Beijing family (*p* < 0.05 represents a statistically significant difference).

The total drug resistance rate of the new cases were significantly less than that of the retreatment patients, and the same results were shown in INH, RIF,SM and EMB mono-resistance rate and MDR rate between the two groups (Table 4). In this study, we analyzed the genotypes and the association relationship between drug resistance and Beijing genotype family strains. However, the Beijing genotype and non-Beijing genotype family didn’t show any statistical associations in any drug resistance frequency (Table 4).

Statistic results also showed no significant difference between different genotype when compared in pan-susceptible TB and any drug resistant TB groups (Table 5). These results indicated that there was no statistical association relationship between Beijing genotype strains and the drug resistance of the 4 kinds of first-line anti-tuberculosis drugs.

**Table 5:** The General demographic characteristics difference of Beijing genotype family and non-Beijing family patients among the pan-susceptible TB and any drug resistant TB.

Characteristic	Category	Pan-susceptible TB					Any drug resistant TB				
		Number of reported cases	Beijing family (%)	Non-Beijing family (%)	OR (95% CI)	*p* value	Number of reported cases	Beijing family (%)	Non-Beijing family (%)	OR (95% CI)	*p* value
All		172	138	34			96	81	15		
sex	Male	132	107(77.5)	26(76.5)	1.01(0.82–1.25)	0.447	74	63(77.8)	11(73.3)	1.06(0.76–1.47)	0.35
	Female	40	31(22.5)	8(23.5)			22	18(22.2)	4(26.7)		
Age, years	<44	108	89(64.5)	19(55.9)	1.15(0.84–1.59)	0.176	58	48(59.3)	10(66.7)	0.89(0.59–1.32)	0.254
	≥44	64	49(35.5)	15(44.1)			38	33(40.7)	5(33.3)		
History of TB	New cases	154	124(89.9)	30(88.2)	1.02(0.89–1.16)	0.391	53	45(55.6)	8(53.3)	1.04(0.62–1.73)	0.487
	Retreated case	18	14(10.1)	4(11.8)			43	36(44.4)	7(46.7)		
household	city	83	68(49.3)	15(44.1)	0.12(0.73–1.69)	0.295	64	53(65.4)	11(73.3)	0.89(0.63–1.25)	0.275
	migrant population	89	70(50.7)	19(55.9)			32	28(34.6)	4(26.7)		

*p* value indicates whether there is a significant difference between Beijing family and non-Beijing family. (*p* < 0.05 represents a statistically significant difference).

## Discussion

In Beijing, a super megacity, the capital of the People’s Republic of China, TB is a reemerging infectious disease and a substantial public health problem^17,19^. Beijing genotype family is the most successful lineage in the present tuberculosis epidemic of Beijing. However, there has been no systematic study on the association between drug resistance and Beijing genotype. So, it is very necessary to study the molecular epidemiology and the association with drug-resistance.

Similar with the results of different study in China^16^, our data demonstrated that Beijing genotype was the most prevalent genotype in Beijing. Based on the Spoligotyping and VNTR data, an interesting finding of this study was the higher clustering rate of Beijing genotype compared with non-Beijing genotype (Table 3). These clustered strains usually suggested the possibility of the recent TB transmission. The result suggested the *M*. *tb* isolates within Beijing genotype family demonstrated a relatively high level of transmission in Beijing. Based on the fact, in order to control TB much better in Beijing, these patients carried clustered strains should be given more attention and investigate their social network in the following study.

In this study, we didn’t found any different risk factors between Beijing and non-Beijing genotype strains, even considered clustered and non-clustered patients (see Supplementary Tables S2 and S3). The spoligotyping and VNTR data give us the same result. So, more study and data were needed to precisely identify the nature of transmission between different genotype strains.

Because some new unknown strains were found out in our study, it is important to check out whether they belong to Beijing genotype or non-Beijing genotype. So we analyzed these data using BioNumerics, we got the MST map. According to the MST map, the newfound strains were belonged to the subordinate of non-Beijing genotype. These strains were firstly found and reported in the world, and deeply study should be carried on in the future.

In previous study, genotyping studies were implemented mainly focused on the distribution of *M*. *tb* genotypes in Beijing^11^. Studies on the association relationship between strains genotype and drug resistance were still insufficient in Beijing. After analyzed the frequency of the Beijing genotype among these strains showing different drug resistance, we found the drug-resistant rate of Beijing genotype strains were not higher than that of non-Beijing genotype strains. The result was not consistent with several publications in other regions of the world. Those studies considered that Beijing genotype was associated with drug resistance and those strains showed higher drug resistance level^20,21^. Meanwhile, other studies also showed no clearly association relationship between drug resistance and strains clade^13,22^.

Different results obtained from different studies and different regions possibly because of the amount of Beijing genotype sublineages and the percentage of modern or ancient Beijing genotype, which affected the DST results^22^. In 2006 Mokrousov *et al*. hypothesized the role of Beijing sublineages that affected DST result which better explained the variability of situations in different countries and districts^23^. Mathuria JP *et al*. also concluded that Beijing genotype strains were not so common in north India and these strains were not fully associated with MDR^24^. Many recent researches also demonstrated the complex DST profiles of Beijing genotype^25,26^. Secondly, the difference among these patterns might be related to the variation in treatment regimens, compliance to treatment protocols, and varying quality of drugs^27^. The association relationship between genetic diversity and DST of *M*. *tb* is deserved to study intensively in the future.

This study suffered from several limitations. The primary limitation was that the amount of isolated strains was not abundant, which may affect the real association. In order to match the results of genotyping and the results of DST each other, we had to move some samples out. We would furtherly validate the result with larger sample size isolated strains in the future. Secondly, the DST experiments only included the first line anti-TB drugs. The first line anti-TB drugs still were the most common used for initial treatment TB. When this study was carried out, we mainly wanted to clarify the association between them and genotype prevalent. We will investigate all the anti-tuberculosis drugs used in clinical at present in the future. In addition, although 12-locus VNTR typing method was used in this study, but our previous results indicated that this 12-locus VNTR method gives appropriate results and HGDI for genotyping in Beijing, which had almost equivalent discriminatory power to that of the 15-locus or 24-locus VNTR for TB genotyping^18^, especially when applied to Beijing family strains. So in this study, 12-locus VNTR method was used again.

In summary, these results suggested there was no statistical association relationship between Beijing genotype family strains and the drug resistance of the four first-line anti-tuberculosis drug. This study also offered a correlation picture of drug resistance and strains genotype in Beijing. We will continue to pursue the study of the association relationship between genotype diversity and drug resistance. These work will be helpful to gain a better understanding of the epidemiology of *M*. *tb* strains and to improve the TB prevention and control in Beijing.

## Methods

### Study setting and design

*M*. *tb* isolate strains obtained from pulmonary TB patients, whose sputum were culture-positive for *M*. *tb*, were delivered to the Beijing Tuberculosis and Thoracic Tumor Research Institute and then were frozen. For any patient with multiple isolates from the culture, only the first isolate was used for performing the bacterial characterization. Six hundred and seventy-nine strains were collected randomly from Beijing from June to December in 2008, which was believed to possess representative and scientific meanings. The strains were recovered from −80 °C stock and were subcultured on solid Lowenstein–Jensen (LJ) medium at 37 °C for three to four weeks. The clinical information of the enrolled patients were retrospectively reviewed and collected, including sex, age, household residence and history of treatment, *et al*. Informed consent was obtained from all subjects included in the study. The protocols and procedures for the protection of human subjects were approved by the Ethics Committee of Beijing Chest Hospital. Furthermore, all the methods were carried out in accordance with the approved guidelines.

### Spacer oligonucleotide typing (spoligotyping)

Spoligotyping was used to identify TB strains genotype according to DR locus as described previously^28^. Typical Beijing genotype strains were defined with the pattern that hybridized to all of the last nine spacer oligonucleotides (spacers 35 to 43), and Beijing-like genotype strains were the ones that hybridized to only some of the last nine spacers. Both typical Beijing and Beijing-like genotype are Beijing genotype. Genomic DNA was extracted by boiling the freshly cultured bacteria as previously described^29^, Spoligotyping was carried out based on the standard protocol^30^. A commercially available kit (Isogen Bioscience BV, Maarssen, and the Netherlands) was used as described by the manufacturer. The results were compared with the SITVIT_WEB database (an international spoligotype database at the Institute Pasteur de Guadeloupe. http://www.pasteur-guadeloupe.fr:8081/SITVIT_ONLINE/)^31^

### VNTR typing

12-locus VNTR combination was used in this study, which same to the previous report^18^. The PCR products were analyzed by electrophoresis in 2% agarose gels, the number of repeats at each locus was interpreted based on the electrophoretic mobility of the products compared with the standard molecular marker (50 bp DNA ladder, TaKaRa-Bio Inc., Dalian, China). H37Rv and double-distilled H_2_O were used as positive and negative controls, respectively. The copy number of repeats was calculated using the following formula: (length of the PCR product minus length of the flanking regions)/length of one repeat copy unit. Alleles were assigned numerical values according to the number of repeats present at a specific genomic locus. Strains were classified as different genotypes according to VNTR loci set while strains having identical VNTR profile type were defined as a cluster. VNTR dendrograms were constructed by using unweighted pair group method with arithmetic averages (UPGMA).

### Drug susceptibility testing (DST)

Mycobacterium tuberculosis drug susceptibility testing (DST) to four first-line anti-TB drugs (Isonazid, Rifampin, Ethambutol, and Streptomycin) was performed using proportion method, as recommended by WHO/IUATLD^32^. Strains which showed resistance to Isonazid and Rifampin were defined as MDR-TB. Strains declared resistant to drugs when growth rate exceeded 1% compared to the control strain. All drugs were purchased from Sigma-Aldrich (St. Louis, MO).

### Data management

Genotyping results showed with binary and octal formats in Microsoft Excel spreadsheets. All spoligotyping data were submitted to the SITVIT_WEB database (http://www.pasteur-guadeloupe.fr:8081/SITVIT_ONLINE/). VNTR data expressed in decimal format were analyzed. Resulting data was analyzed by BioNumerics (Version 5.0, Applied Maths, Sint-Martens-Latem, Belgium) software. Cluster analysis was performed and a dendrogram was generated in Bionumerics using the Dice similarity coefficient and UPGMA coefficient.

The discriminatory power (the Hunter-Gaston discriminatory index (HGDI)) of each typing method was calculated according to a previously published method^33^:

$$HGDI=1-(\frac{1}{N(N-1)}\sum _{j=1}^{s}{n}_{j}({n}_{j}-1))$$

where *N* is the total number of isolates in the typing method, *s* is the number of distinct patterns discriminated by VNTR, and *n*_*j*_ is the number of isolates belonging to the *j*th pattern. The percentage clustering was calculated with the following formula: (*n*_*c*_ − *C*)/*N*, where *N* is the total number of isolates, *C* is the number of clusters, and *n*_*c*_ is the total number of clustered isolates^34^.

### Statistical analysis

Statistical analysis of genotype and phenotype were performed in SPSS 21.0 software. Chi-square test or Fisher’s exact probability test was used to compare the proportions of different groups. A *p* value less than 0.05 was considered statistically significant. Odd ratios (ORs) and 95% confidence intervals (CI) were calculated to measure the association relationship between genotype and drug susceptibility results.

### Availability of data and materials

The online version of this article contains supplementary material, which is available to authorized users.

## Electronic supplementary material

Supplemental Information

Supplementary Dataset File
