# Supplementary material for: The study on the association between Beijing genotype family and drug susceptibility phenotypes of Mycobacterium tuberculosis in Beijing
Source: Sci Rep. 2017 Nov 8;7:15076. doi: 10.1038/s41598-017-14119-z (PMC5678160; doi:10.1038/s41598-017-14119-z)
Supplement: Supplementary file 1 — Supplemental Information [file 41598_2017_14119_MOESM1_ESM.pdf]

The study on the association between Beijing genotype family and drug susceptibility phenotypes of *Mycobacterium tuberculosis* in Beijing.

Yi Liu[1#]*, Xiaoying Jiang[1#], Wensheng Li[1], Xuxia Zhang[1], Wei Wang[1], Chuanyou Li[1] *

1 Department of Bacteriology and Immunology, Beijing Key Laboratory on Drug-Resistant Tuberculosis Research, Beijing Tuberculosis and Thoracic Tumor Research Institute/Beijing Chest Hospital, Capital Medical University, Tongzhou District, Beijing, 101149, PR China.

# These authors contributed equally to this work.

***Corresponding author's contact information:**

Correspondence should be addressed to Chuanyou Li (lichuanyou6688@hotmail.com) or Yi Liu (liuyilotus@hotmail.com).

Mailing address: NO.9 BeiGuanDaJie, Tongzhou District, Beijing, 101149, China.

Tel: 8610-89509367; Fax: 8610-69546819.

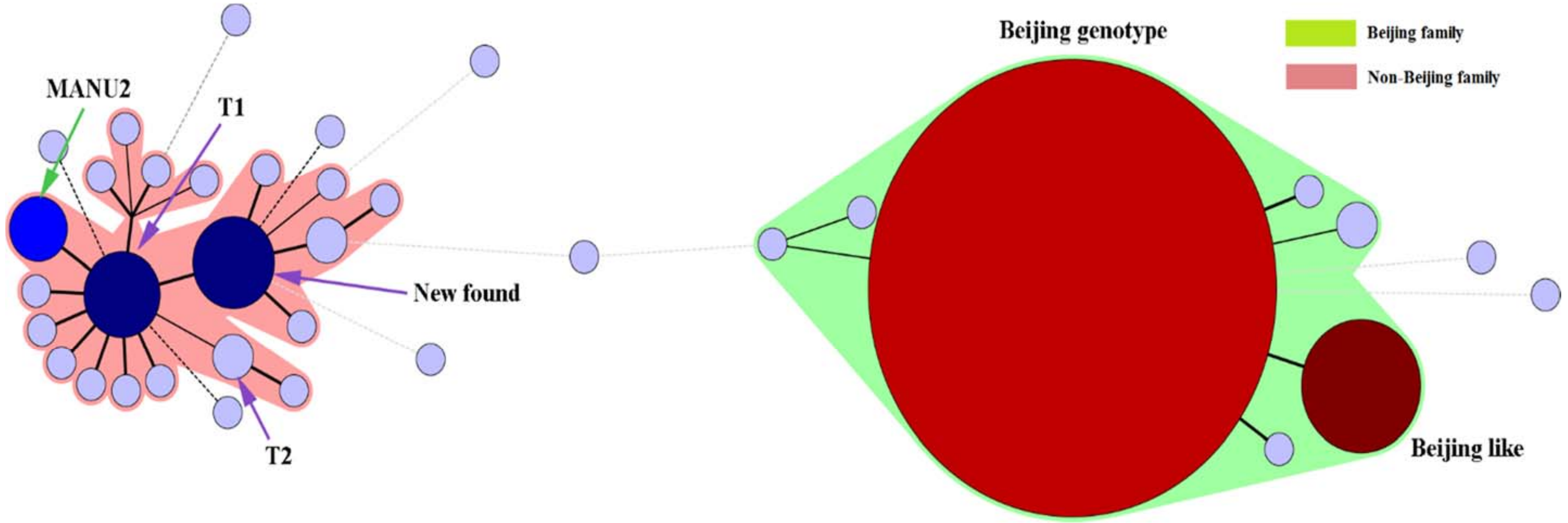

Fig. S1. Minimum spanning tree showing the clustering by spoligotyping of 268 *M. tuberculosis* strains in Beijing.
Each nodal point represents a particular spoligotype, and the size of each nodal point is relative to the number of strains within that spoligotype.
The annotations in the figure were the most frequent spoligotypes.

Table S1. Spoligotyping patterns result of *M. tuberculosis* strains collected from Beijing in this study.

No.$	% (No.)										
	**Beijing families***		**non-Beijing families#**								
	Typical Beijing	Atypical Beijing	T1	T2	T5	MANU 2	MANU 3	H3	EAI1_SOM	Cas1-Delhi	New found
268	74.2% (198)	7.9% (21)	6% (16)	1.9% (5)	1	2.6% (7)	1	1	1	1	5.29% (16)

$ The number of collected strains isolates.

* 219(81.7%) strains belonged to Beijing families.

49(18.3%) strains (include 16 newfound strains) belonged to non-Beijing families.

Table S2. Cluster analysis of patients (n=268) merged with background information after spoligotyping analyzing.

Characteristic	Variable	Number of reported cases (%)	Number of clustered patients (%)	Number of non-clustered patients (%)	OR (95% CI)	*p* Value
All		268	240	28		
Sex	Male	206(76.9)	189(78.8)	18(64.3)	1.225(0.922-1.627)	0.072
	Female	62(23.1)	51(21.2)	10(35.7)		
Age groups, years	<44	166(61.9)	146(60.8)	20(71.4)	0.852(0.660-1.099)	0.188
	≥44	102(38.1)	94(39.2)	8(28.6)		
Household	City	147(54.9)	134(55.8)	13(46.4)	1.203(0.795-1.818)	0.227
	migrant population	121(45.1)	106(44.2)	15(53.6)		
History of TB	No Treatment	207(77.2)	184(76.7)	25(89.3)	0.859(0.742-0.994)	0.094
	Treatment	61(22.8)	56(23.3)	3(10.7)		

$p<0.05$ represents a statistically significant difference

Table S3. Cluster analysis of patients (n=268) merged with background information after VNTR analyzing.

Characteristic	Variable	Number of reported cases (%)	Number of clustered patients (%)	Number of non-clustered patients (%)	OR (95% CI)	*p* Value
All		268	112	156		
Sex	Male	206(76.9)	85(75.9)	121(77.6)	0.978(0.856-1.119)	0.430
	Female	62(23.1)	27(24.1)	35(22.4)		
Age groups, years	<44	166(61.9)	65(58)	101(64.7)	0.896(0.737-1.090)	0.162
	≥44	102(38.1)	47(42)	55(35.3)		
Household	City	147(54.9)	58(51.8)	88(56.4)	0.918(0.733-1.151)	0.266
	migrant population	121(45.1)	54(48.2)	68(43.6)		
History of TB	No Treatment	207(77.2)	91(81.2)	116(74.4)	1.093(0.961-1.242)	0.119
	Treatment	61(22.8)	21(18.8)	40(25.6)		

$p<0.05$ represents a statistically significant difference
